# Comparison of Physiological Parameters and Anaesthesia Specific Observations during Isoflurane, Ketamine-Xylazine or Medetomidine-Midazolam-Fentanyl Anaesthesia in Male Guinea Pigs

**DOI:** 10.1371/journal.pone.0161258

**Published:** 2016-09-22

**Authors:** Sabrina Schmitz, Sabine Tacke, Brian Guth, Julia Henke

**Affiliations:** 1 Department of Nonclinical Drug Safety, Biological Laboratory Service, Boehringer Ingelheim Pharma GmbH & Co. KG, Biberach, Germany; 2 Department of Veterinary Clinical Sciences, Clinic for Small Animal-Surgery, Justus-Liebig-University, Giessen, Germany; 3 Department of Drug Discovery Support, General Pharmacology, Boehringer Ingelheim Pharma GmbH & Co. KG, Biberach, Germany; 4 DST/NWU Preclinical Drug Development Platform, Faculty of Health Sciences, North-West University, Potchefstroom, South Africa; Hopital Robert Debre, FRANCE

## Abstract

Guinea pigs (GPs) are difficult to anaesthetize successfully, the choices for anaesthesia are limited and physiological parameters are likely to be influenced substantially under anaesthesia. We implanted blood pressure radio-telemetry devices into 16 male GPs and subjected them to anaesthesia with ketamine-xylazine (KX), medetomidine-midazolam-fentanyl (MMF) or isoflurane (Iso, plus atropine premedication) in a randomized order with a 7 day interval between anaesthesias. Each anaesthesia lasted 40min, after which Iso was discontinued, MMF was fully antagonized with atipamezole-flumazenil-naloxone and KX was partially antagonized with atipamezole. Hemodynamics were recorded continuously for at least 240min after induction and the GPs were monitored for respiratory rate, reflex responses and specific observations until regaining of their righting reflex (RR). Blood for glucose testing was taken from the ear at 7.5, 20 and 40min during anaesthesia. Recovery time was short with MMF and Iso but long for KX. MMF induced only a transient blood pressure drop after antagonization, whereas Iso caused a marked hypotension during maintenance and KX led to moderate hypotension after antagonization. MMF and Iso produced tolerable heart rate changes, but KX led to long term post-anaesthetic bradycardia. Hypothermia occurred with all anaesthesias, but the GPs returned to normothermia the fastest under MMF, followed shortly by Iso. KX, however, caused a profound and prolonged hypothermia. The respiration was depressed with all anaesthesias, substantially with MMF (-41%) and KX (-52%) and severe during Iso maintenance (-71%). Blood glucose with MMF and KX increased throughout the anaesthesia, but the values remained within reference values with all anaesthetics. The reflex responses character and strength varied between the anaesthetics. In conclusion, MMF is the anaesthetic of choice and Iso may be used for short, non-painful procedures. We advise against the use of KX in GPs.

## Introduction

Anaesthesia is regularly required in guinea pigs (*Cavia porcellus*, GPs). They are considered to be one of the most difficult rodents to anaesthetize safely [1], and only few anaesthetics show satisfactory effect [2]. It is known that anaesthesia significantly alters physiological parameters [3], but so far in GPs its’ influence has not been investigated thoroughly. We implanted radio-telemetry devices having a blood pressure transducer into male GPs and continuously measured the effect of 3 commonly used anaesthetics during anaesthesia induction, maintenance and recovery. We investigated the influence on hemodynamic parameters, core body temperature, respiratory rate (ReR), blood glucose, reflex responses and anaesthesia-related observations.

## Materials and Methods

### Housing and acclimatization

This research was approved by the Regierungspräsidium Tübingen, Germany under the approval number 12–038. For anaesthesia isoflurane, the combination of medetomidine-midazolam-fentanyl and ketamine-xylazine were used. Euthanasia was performed with pentobarbital. All experiments and procedures were performed in accordance with the German Animal Welfare Act (Art. 3 G v. 28.7.2014 I 1308) and the regional council for animal welfare.

Sixteen male albino Hartley guinea pigs from Charles River Laboratories (Sulzfeld, Germany, delivery body weight (BW) 350-400g/ age of 6.5 weeks) were housed for 19 days prior to the radio-telemetry implantation in groups of 2–3 (EHRET TERULAN THF 1776) with wooden bedding material (Lignocel FS14, Rettenmaier & Söhne GmbH + Co.KG, Rosenberg, Germany, change 2x/week). Two shelters per cage, 20g/GP of pelleted diet (3410 complete feed, KLIBA NAFAG, Provimi Kliba Sa., Kaiseraugst, Switzerland) and a large amount of autoclaved hay were supplied daily. Tap water was available *ad libitum*. The animal room was kept at 20±2°C, 55±10% and had an air change of 15 cycles/h. Light-dark cycle was 12:12 ± dimmer phases of 30min. A radio played music for acoustic habituation when lights were on. BW and general condition of each animal were monitored daily. The animals were acclimatized daily to being handled and to being single housed.

### Radio-telemetry transmitter implantation

The perioperative antibiotic and analgesic medication with enrofloxacin (10mg/kg, Enrotron 100mg/mL, aniMedica GmbH, Senden-Bösensell), meloxicam and (0,4mg/kg, Metacam, Boehringer Ingelheim Vetmedica GmbH, Ingelheim, Germany) and metamizole (80mg/kg, Novalgin, Sanofi-Aventis Deutschland GmbH, Frankfurt am Main, Germany), as well as the implantation of the radio-telemetry transmitter (DSI, PhysioTel HD, HD-S11, DSI, St.Paul, MN, USA) was performed as described previously [4]. The surgery was carried out under MMF anaesthesia [5] (medetomidine 0.2mg/kg, midazolam 1.0mg/kg, fentanyl 0.025mg/kg, see Table 1) and the same dosage was also used for the following MMF anaesthesias.

**Table 1:** Medication used during radio-telemetry transmitter implantation and for MMF anaesthesia in male guinea pigs.

Purpose	Product name	Brand name / Manufacturer
anaesthesia- agonists	Intramuscular (i.m.) in mixed syringe MMF = 1. Medetomidine^1^ 0,2mg/kg +2. Midazolam^2^ 1,0mg/kg + 3. Fentanyl^3^ 0,025mg/kg	1. DOMITOR, 1mg/mL, Orion Corporation, Espoo, Finland. 2. Dormicum 5mg/mL, Roche Pharma AG, Grenzach-Wyhlen, Germany. 3. Fentanyl-Janssen 0.1mg/2mL, JANSSEN-CILAG, Neuss, Germany
anaesthesia- antagonists	Subcutaneous (s.c.) in mixed syringe AFN = 4. Atipamezole^4^ 1,0mg/kg +5. Flumazenil^5^ 0,1mg/kg + 6. Naloxone^6^ 0,03mg/kg	4. ANTISEDAN 5mg/mL, Orion Corporation, Espoo, Finland. 5. Flumazenil HEXAL 0.1mg/mL, HEXAL AG, Holzkirchen, Germany. 6. Naloxon Inresa 0,4mg/mL, Inresa Arzneimittel, Freiburg, Germany

### Study design

Thirteen GPs were each subjected to anaesthesia with Iso (Forene100%, AbbVie Deutschland, Ludwigshafen, Germany), KX (Ketasol -100 ad us.vet., Dr. E. Graeub AG, Bern, Switzerland; Rompun 2% Bayer Vital GmbH, Leverkusen, Germany) or MMF using a randomized cross-over design with a 7 day interim between the anaesthesias (day 0, 7, 14). BP, HR and BT were continuously measured via radio-telemetry. A maximum of 2 GPs per day were anaesthetized sequentially. Baseline values for both GPs were acquired before the first anaesthesia started and the GPs were premedicated at 10min before induction. After 40min, MMF was antagonized with AFN, KX was partially antagonized with atipamezole (0.15mg/kg ANTISEDAN, Orion Corporation, Espoo, Finland) and Iso exposure was stopped. A total measuring cycle consisted of 2h habituation (the last 15min were used as the baseline values), 10min premedication, 40min of anaesthesia and wake-up and recovery phases as long as needed for each individual. Data was acquired for at least 240min after induction. All procedures were done by the same veterinarian.

### Experimental Protocol

The GPs were weighed, examined (condition of fur, eyes, nose, posture and behaviour) and each placed into a single cage (Makrolon type III, EHRET Labor- und Pharmatechnik, Emmendingen, Germany) equipped like the home cage. They were transported to the data acquisition room with lighting, climate and radio settings the same as in the home cage room. Telemetry receiver plates were covered with a heating mat, one cage was placed on top and the data acquisition was started. After 2h of alone habituation time, the first GP was lifted out of its cage and premedicated in the dorsal neck (see 2.5–2.7). The heating mat was turned on (39°C) until the GP had recovered to a BT of at least 38°C. Anaesthesia was induced (see detailed description for each treatment below), the GP was placed on its back on the heating mat and eye lubricant (VitA-Pos, URSAPHARM, Saarbrücken, Germany) was administered after loss of the righting reflex (RR). Pure oxygen (0.7mL/min) was supplied through a nose cone from loss of RR to antagonization, after which the GP was replaced to its single cage in dorsal recumbency to assess the time until RR returned. During anaesthesia, reflex responses (see Table 2) and ReR (visual assessment of breaths per minute) were evaluated in 2.5min intervals from 0-15min and 40-55min and in 5min intervals from 15-40min (0min = induction) or until the RR was regained (10min interval from 60-…min).

**Table 2:** Reflex tests, observations and responses during guinea pig (GP) anaesthesia.

Name	Definition for positive response
Righting reflex (RR)	The GP is able to right itself when placed on its back
Lid response (LR)	Blink to touching of the eyelid
Ear Reflex (ER)	Ear twitching to a skin touch of the ear canal entrance
Foot withdrawal reflex (FWR)	The GP withdraws its extended hind foot to a fingernail pinch on one toe
Inguinal reflex (IR)	The GP kicks a hind leg, when pinched up to the first clamp catch in the inguinal region lateral to the nipples
Muscle tone	Evaluation of counter pull to hind leg extension
Chewing	Chewing movements without food or water uptake
Shivering	Rhythmical torso and lower back muscle contractions
Piloerection	Ruffled instead of sleek fur
KX only: sedation	Head drooping, staggering walk, reduced responsiveness, squinted eyes, delayed reflexes
Iso only: Cleaning, eye watering, blinking	Tearing eyes, squinting/blinking, fore feet rubbing over the ears and nose, anogenital region licking
Iso only: respiratory sounds	Breathing related rattle, coughing over the trachea and lung, audible with or without stethoscope
Skin colour	Evaluated on the eye lids, ears, lips, front feet and hind feet

Reflex responses were rated from–to +++. No reaction was considered a negative (-) result, a minimal response tested ±, a mild one as +, a delayed/reduced response as ++ and a physiological response as +++. Only–and ± responses to FWR and IG were considered surgically tolerant. Hectic movements, running and restlessness were defined as excitation. Blood glucose (BG) was measured during the anaesthesia (OneTouch Ultra2, LifeScan Europe, Zug, Switzerland) at 7.5, 20 and 40min, with blood taken from an ear prick with a blood lancet (Solofix B.Braun, Melsungen, Germany). In case of apnoea, the GP was tilted back and forth for ventilation and was replaced to the nose cone after regular breathing was restored. GP was warmed via water filled gloves, if BT dropped below 35°C. The depth of anaesthesia was divided into reflex-dependent intervals (Table 3).

**Table 3:** Reflex dependent anaesthesia interval definition for anaesthesia with MMF, isoflurane and KX in male guinea pigs.

Anaesthesia interval	Definition
I = induction	From MMF, KX injection or isoflurane exposure to loss of RR
II = non-surgical tolerance	From loss of RR to mildly positive (±) foot withdrawal and inguinal reflex
III = surgical tolerance	From (±) foot withdrawal and inguinal reflex to (partial) antagonization
IV = wake-up	From (partial) antagonization to regaining of RR
V = recovery	From RR until 240min

For reflex definition see Table 2. RR = righting reflex, MMF = medetomidine-midazolam-fentanyl, KX = ketamine-xylazine.

NOTOCORD-hem was used for the telemetric data acquisition. Values between premedication (-10min) until 60min after induction were averaged in 20sec intervals and thereafter in 2.5min intervals.

No statistical evaluation was done. Baseline and anaesthesia values are presented descriptively (Tables 4, 5 and 6).

**Table 4:** Baseline and anaesthesia values during medetomidine-midazolam-fentanyl (MMF), isoflurane (Iso) and ketamine-xylazine (KX) anaesthesia in male guinea pigs.

Parameter	Treat-ment	N	Baseline	Induction	Non-surgical tolerance	Surgical tolerance	Wake-up	Recovery
SAP	MMF	11	66.9 ± 2.7	79.0 ± 12.4	69.5 ± 15.6	68.5 ± 13.2	39.8 ± 7.7	72.2 ± 3.6
[mmHg]	Iso	13	66.3 ± 5.0	91.9 ± 8.6	43.0 ± 11.2	20.6 ± 7.7	47.3 ± 11.9	70.4 ± 5.7
	KX	7	68.3 ± 3.4	78.1 ± 10.7	61.2 ± 14.6	66.0 ± 8.6	50.6 ± 6.4	61.5 ± 4.1
DAP	MMF	11	48.1 ± 2.7	57.0 ± 7.9	51.2 ± 10.0	50.3 ± 8.6	28.8 ± 5.5	52.3 ± 3.1
[mmHg]	Iso	13	48.4 ± 3.8	67.4 ± 7.1	32.2 ± 8.0	16.4 ± 6.7	35.4 ± 9.2	50.6 ± 3.5
	KX	7	48.5 ± 4.7	55.1 ± 7.5	44.2 ± 10.0	47.7 ± 5.8	37.5 ± 4.5	47.9 ± 4.3
MAP	MMF	11	57.6 ± 3.3	67.4 ± 9.7	59.8 ± 12.5	58.8 ± 10.8	34.3 ± 6.5	62.0 ± 3.2
[mmHg]	Iso	13	56.9 ± 3.7	78.8 ± 7.8	37.6 ± 9.6	18.5 ± 7.2	41.4 ± 10.4	60.2 ± 4.4
	KX	7	58.9 ± 3.2	66.0 ± 9.1	52.4 ± 12.2	56.1 ± 7.2	43.8 ± 5.6	54.1 ± 4.1
HR	MMF	11	245.8 ± 18.2	277.3 ± 25.4	233.5 ± 8.2	214.2 ± 10.0	240.6 ± 20.7	288.4 ± 20.8
[bpm]	Iso	13	247.0 ± 16.0	291.8 ± 29.1	275.2 ± 20.9	244.9 ± 16.0	278.0 ± 15.8	284.0 ± 10.9
	KX	7	237.9 ± 11.1	263.3 ± 20.9	218.4 ± 9.0	197.0 ± 7.8	206.8 ± 28.9	212.1 ± 9.5
BT	MMF	11	38.9 ± 0.2	38.6 ± 0.3	38.3 ± 0.4	37.0 ± 0.5	36.3 ± 0.7	38.6 ± 0.6
[°C]	Iso	13	38.9 ± 0.4	38.9 ± 0.4	38.7 ± 0.4	37.0 ± 0.4	35.8 ± 0.6	38.5 ± 0.3
	KX	7	39.0 ± 0.2	38.6 ± 0.3	38.4 ± 0.4	37.5 ± 0.3	36.6 ± 0.7	37.5 ± 0.7

Mean and SD values, baseline = average of 15min before induction of anaesthesia, SAP = systolic arterial pressure, DAP = diastolic arterial pressure, MAP = mean arterial pressure, HR = heart rate, BT = core body temperature.

**Table 5:** Respiratory rate during anaesthesia with medetomidine-midazolam-fentanyl, isoflurane and ketamine-xylazine in male guinea pigs.

**Induction**	**2.5min**		**5min**		**7.5min**		**10min**		**12.5min**		**15min**	
**Treatment**	**n**	**mean ± sd**	**n**	**mean ± sd**	**n**	**mean ± sd**	**n**	**mean ± sd**	**n**	**mean ± sd**	**n**	**mean ± sd**
MMF	10	83.2 ± 14.5	11	73.5 ± 9.3	11	71.6 ± 9.0	11	67.7 ± 9.9	11	71.3 ± 13.1	11	69.5 ± 12.0
Iso	12	92.0 ± 18.8	13	76.9 ± 13.3	13	65.5 ± 11.1	13	50.5 ± 16.9	13	42.9 ± 11.4	13	37.2 ± 11.0
KX	6	61.3 ± 19.0	7	56.6 ± 6.7	7	57.1 ± 7.2	7	56.6 ± 4.3	7	55.4 ± 3.6	7	57.7 ± 5.1
**Maintenance**	**20min**		**25min**		**30min**		**35min**		**40min**			
**Treatment**	**n**	**mean ± sd**	**n**	**mean ± sd**	**n**	**mean ± sd**	**n**	**mean ± sd**	**n**	**mean ± sd**		
MMF	11	70.2 ± 12.9	11	69.5 ± 14.3	11	67.6 ± 13.0	11	66.5 ± 13.1	11	65.8 ± 12.3		
Iso	13	34.9 ± 13.2	13	31.4 ± 10.8	13	32.6 ± 12.4	13	28.9 ± 12.7	13	37.4 ± 14.5		
KX	7	56.0 ± 4.6	7	55.4 ± 6.3	7	54.9 ± 6.0	7	54.9 ± 6.0	7	56.6 ± 4.9		
**Recovery**	**42.5min**		**45min**		**47.5min**		**50min**		**52.5min**		**55min**	
**Treatment**	**n**	**mean ± sd**	**n**	**mean ± sd**	**n**	**mean ± sd**	**n**	**mean ± sd**	**n**	**mean ± sd**	**n**	**mean ± sd**
MMF	11	64.0 ± 17.3	11	63.3 ± 14.6	10	89.2 ± 23.9	11	93.5 ± 18.9	11	90.2 ± 18.0	9	91.6 ± 13.6
Iso	13	53.2 ± 9.9	13	60.6 ± 10.2	13	62.2 ± 11.5	13	71.1 ± 16.8	12	75.3 ± 17.7	12	76.7 ± 13.6
KX	7	49.7 ± 5.6	7	48.6 ± 7.1	7	44.0 ± 8.9	7	41.7 ± 8.6	7	41.7 ± 8.6	7	42.9 ± 5.5
	**60min**		**65min**		**70min**		**80min**					
**Treatment**	**n**	**mean ± sd**	**n**	**mean ± sd**	**n**	**mean ± sd**	**n**	**mean ± sd**				
KX	7	53.1 ± 14.4	7	50.3 ± 14.2	7	62.3 ± 20.4	7	65.7 ± 14.4				

Mean and SD of respiratory rate [1/min], MMF = medetomidine-midazolam-fentanyl, Iso = isoflurane, KX = ketamine-xylazine.

**Table 6:** Blood glucose level during anaesthesia in male guinea pigs.

Unit	Treatment	n	7.5min	20min	Increase [%]	40min	Increase [%]
[mmoL/L]	MMF	11	6.1 ± 1.0	8.7 ± 1.9	+ 41.9	11.1 ± 3.3	+ 27.7
	Iso	13	7.3 ± 1.8	7.7 ± 2.1	+ 5.3	7.9 ± 2.4	+ 2.5
	KX	7	6.2 ± 1.7	8.0 ± 2.2	+ 28.9	10.0 ± 4.1	+ 25.3

Averaged means and SD of blood glucose levels, MMF = medetomidine-midazolam-fentanyl; Iso = isoflurane; KX = ketamine-xylazine.

### MMF anaesthesia

**Fig 1:**
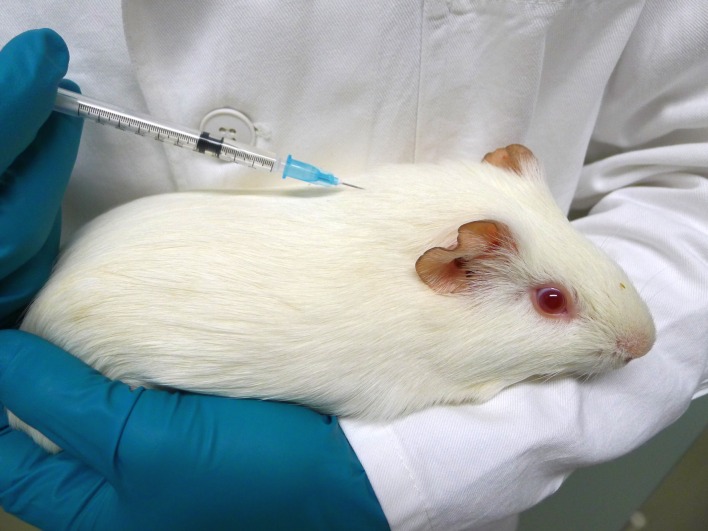
Subcutaneous injection in the neck of a guinea pig.

For premedication 0.4mL/kg of sodium chloride (in this case as a placebo) were injected s.c. into the dorsal neck region (Fig 1).

The GP was immediately returned to the single cage. MMF was mixed in one syringe but the dose was divided, if the total volume exceeded 0.5mL. Injections were given i.m. into the caudal part of the femoral muscle of one/both hind legs and the GP was then returned to its cage. After 40min the antidote AFN was injected s.c.

### Iso anaesthesia

**Fig 2:**
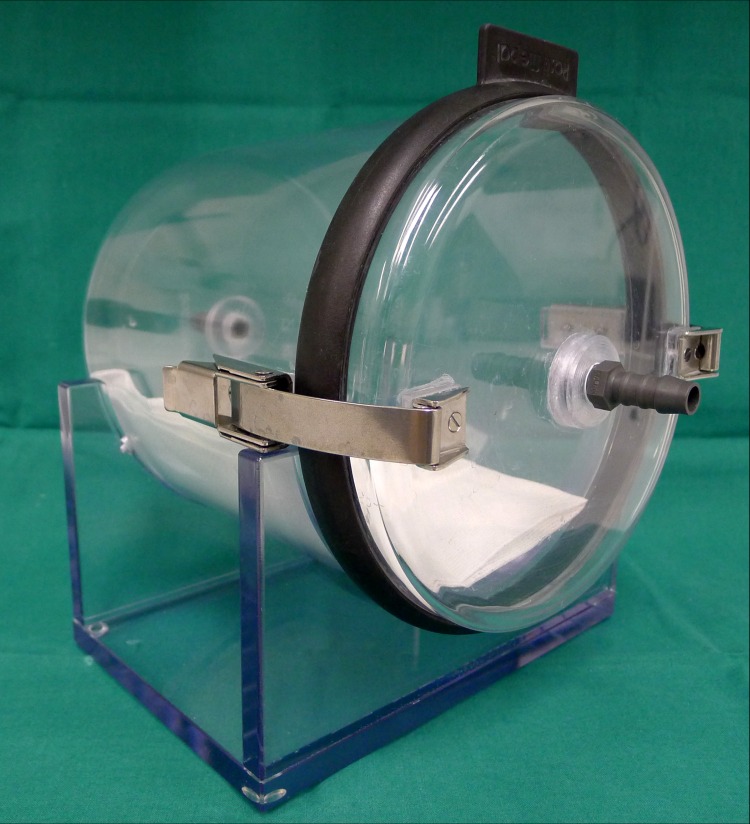
Circular whole body chamber for isoflurane induction in guinea pigs.

Atropine (Atropinum Sulfuricum 1.0mg Eifelfango, Bad Neuenahr-Ahrweiler, Germany) was diluted with sodium chloride to 0.1mg/mL and 0.4mL/kg (0.04mg/kg) of the solution was injected s.c. (as described for MMF). A circular whole body chamber (BC) with an inserted liquid absorbent cloth (Fig 2) was prefilled with 99% O_2_ and 4.4% Iso (measured by Criticare Poet II Patient Monitor, Soma Technology Inc., Bloomfield, USA).

The gas flow was interrupted, the box turned vertical and the GP was lowered, hind legs first, into the chamber. The lid was closed, the BC repositioned horizontally and the gas flow restarted. The GP remained in the BC until loss of RR and of voluntary movement. During the induction in the BC only visual observations could be obtained. Gas inflow was redirected to the nose cone (inflow of 0.7mL O_2_, 3% Iso), the GP was removed from the BC and placed in a dorsal position on the warmed receiver plate with the nose inserted into the nose cone.

### KX anaesthesia

Premedication was the same (sodium chloride) as used with MMF. Ketamine (75mg/kg) and xylazine (15mg/kg) were mixed in a single syringe. For induction, the GP was positioned upright, hind feet touching the ground and KX was injected intraperitoneally into the dorsal abdomen 1cm lateral to the midline. Intraperitoneal application was selected to avoid muscle tissue necrosis and due to the large injection volume. The GP was returned to the single cage until it had lost its RR. At 35min a maximum of 10mL of 39°C warmed 0.9% sodium chloride solution was distributed s.c. over the abdomen to compensate for dehydration. At 40min xylazine was antagonized with 0.15mg/kg atipamezole s.c. into the axillary region.

## Results

Three out of 16 implanted GPs were euthanized before the beginning of the anaesthesia study, 2 because of hind leg lameness at 2 and 6 days after surgery and 1 due to excessive intraoperative bleeding. Thirteen guinea pigs entered the study and all animals survived the anaesthesia study.

With MMF anaesthesia, 2 animals didn’t reach the defined surgical tolerance state (reflex status > ± for FWR and RR) and they were excluded from the hemodynamic data analysis. With KX anaesthesia, 3 animals never lost RR and 3 more didn’t reach surgical tolerance; these 6 animals were also excluded from the hemodynamic evaluation. Consequently, anaesthesia with MMF could be assessed with 11 GPs, 13 for Iso but only 7 GPs for KX. Two GPs showed apnoea under Iso anaesthesia while attached to the nose chamber.

### Anaesthesia duration

**Fig 3:**
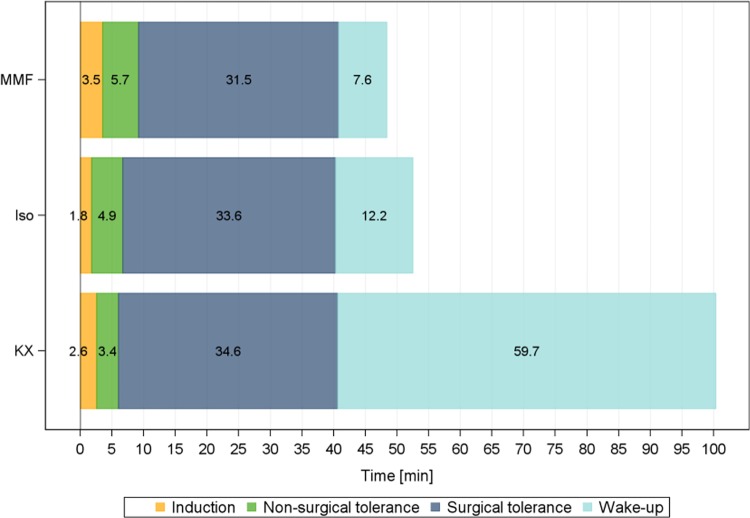
Duration of anaesthesia with MMF, Iso and KX in male guinea pigs. MMF = medetomidine-midazolam-fentanyl, n = 11; Iso = isoflurane, n = 13; KX = ketamine-xylazine, n = 7; interval definition see Table 3.

During anaesthesia induction, non-surgical tolerance and surgical tolerance durations differed only marginally between the 3 anaesthetic regimens (Fig 3).

Induction and non-surgical tolerance were short with a range between 6min for KX and 9.2 min for MMF. Significant differences were seen in the speed until regaining of RR (wake up). MMF had the quickest wake up phase, followed closely by Iso, whereas GPs with KX needed by far the longest time until they regained their RR (59.7min).

### Baseline and anaesthesia values

Baseline values before each anaesthesia were similar apart from a slightly lower HR with KX (238bpm, see (Table 4).

### MMF

**Fig 4:**
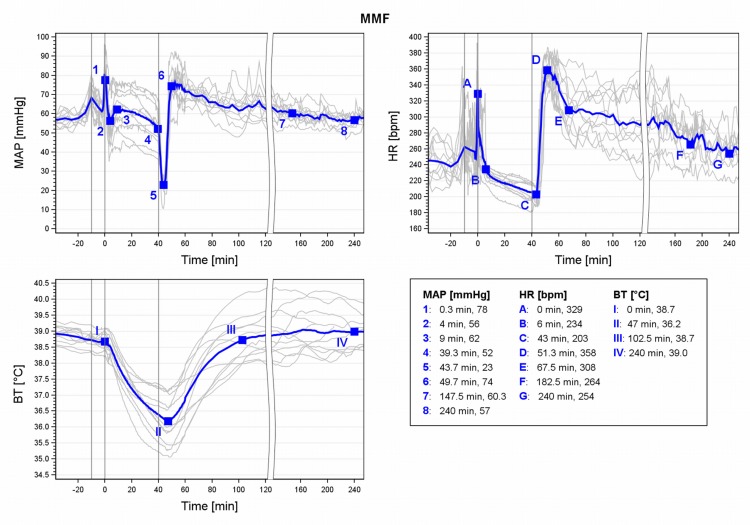
MAP, HR and BT during MMF anaesthesia in male guinea pigs. Mean (blue) and individual arterial pressure (MAP), heart rate (HR) and core body temperature (BT) during medetomidine-midazolam-fentanyl (MMF) anaesthesia in 11 male guinea pigs. 1st grey line ≙ premedication at -10min, 2nd line ≙ induction at 0min; 3rd line ≙ antagonization at 40 min.

During MMF anaesthesia, after a short induction peak in MAP and HR, MAP ranged within baseline values (Fig 4, MAP 3–4) and HR slightly below (Fig 4, HR B-C) during maintenance. Three min after antagonization, the HR increased until 51.3min (mean RR regained at 48.3min). After AFN, MAP resumed baseline levels at 147.5min (7) and HR after 182.5min (F). BT decreased continuously until shortly before RR was regained (Fig 4, BT I-II) and rose from there on to return to baseline (>38.7°C) after 102.5min (III).

### Iso

**Fig 5:**
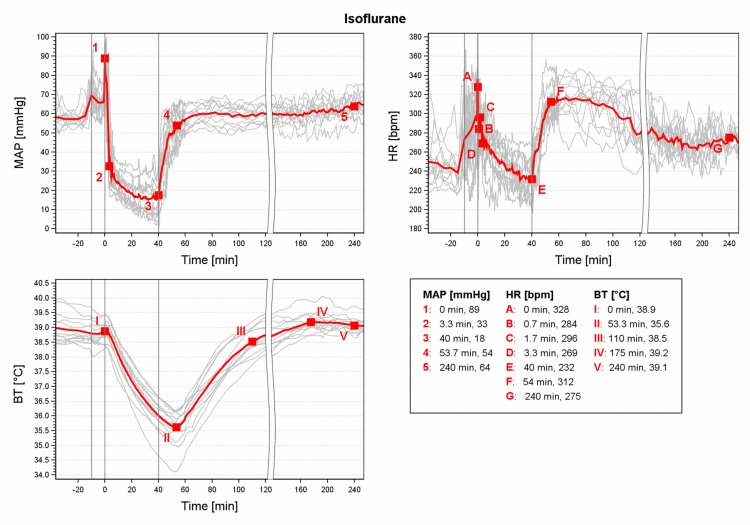
MAP, HR and BT during isoflurane anaesthesia in male guinea pigs. Mean (red) and individual arterial pressure (MAP), heart rate (HR) and core body temperature (BT) during isoflurane anaesthesia in 13 male guinea pigs. 1st grey line ≙ premedication at -10min, 2nd line ≙ induction at 0min; 3rd line ≙ end of exposure at 40 min.

MAP decreased abruptly (-43% to baseline) within 3.3min after induction (Fig 5, MAP 2). The MAP declined even further to very low levels (18 mmHg, -69%) until 40min (3) but increased steeply after Iso exposure was stopped, reaching baseline values at 53.7min (4) and remaining stable thereafter. The HR peaked when placing the GPs into the BC (Fig 5, HR A), but it fell again quickly (B). It slightly rose again, with the transfer to the nose cone (C) then decreased continuously during maintenance to slightly below baseline HR (E). The HR immediately rose after stop of Iso exposure until 54min (F) then decreased continuously but baseline HR values were not reached before 240min (G). BT fell progressively (Fig 5, BT II) until after regaining of RR at 52.5min. It increased after 53.3min and reached stable baseline BT values after 110min (III).

### KX

**Fig 6:**
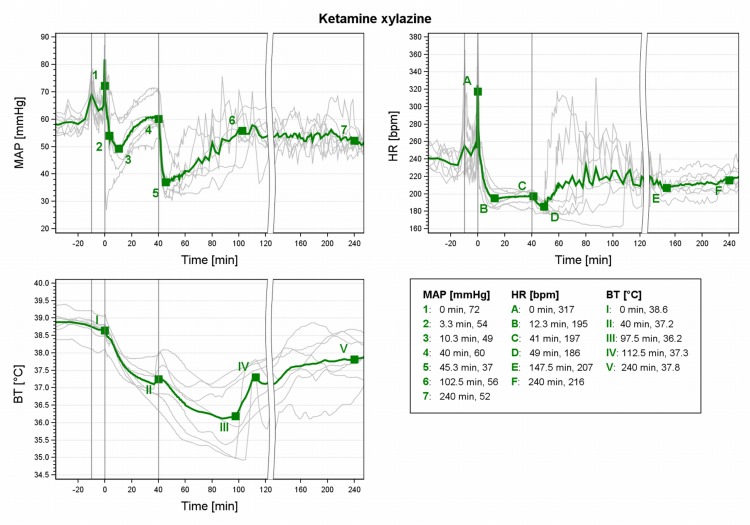
MAP, HR and BT during KX anaesthesia in male guinea pigs. Mean (green) and individual arterial pressure (MAP), heart rate (HR) and core body temperature (BT) during ketamine-xylazine (KX) anaesthesia in 7 male guinea pigs. 1st grey line ≙ premedication at -10min, 2nd line ≙ induction at 0min; 3rd line ≙ partial antagonization at 40 min.

For KX the MAP values varied substantially (Fig 6). During maintenance the MAP increased steadily from slightly below to near baseline values at 40min (4). Atipamezole induced a fall (-39%, 5) followed by a slow advance with wide individual variance. Baseline values were regained at 102.5min (6). The MAP remained stable but slowly decreased to end slightly below baseline MAP. HR dropped after induction until 12.3min (Fig 6, HR B) and remained stable during maintenance. It decreased slightly following atipamezole (D) and thereafter varied widely during recovery until 147.5min (E). From there on the mean HR increased gradually but baseline HR values were not reached until 5.25h after induction (not included in Fig 6). The BT continuously decreased after KX induction (Fig 6, BT I) and it fell further after atipamezole antagonization with individual variance. In 2 GPs the BT dropped below 35°C during recovery and they received external warming. Mean BT gradually increased but all GPs were still hypothermic at 240min after induction (baseline BT 39±0.2°C). Three GPs reached 38.8°C after 5h (307.5, 317.5 and 332.5min), the other 4 GPs had not reached pre-anaesthetic BT levels at 8h.

### Respiration

All drug combinations induced a significant decrease of ReR under anaesthesia (Table 5), which was substantial with MMF (-41%, baseline 114brpm,) and drastic in Iso (-71%). GPs in the BC showed irregular breathing and defence movements during Iso (slowly and deeply → held breath → steadily and more shallow), such that an averaged ReR of the first 2.5min was not representative for that period. ReR was also reduced markedly (-52%) but stably under KX maintenance, yet fell further after the administration of atipamezole. With MMF, the ReR increased rapidly after the GPs had regained their RR, whereas with Iso, ReR progressively increased once the Iso was discontinued.

One GP showed apnoea during Iso maintenance and mild respiratory breathing sounds were auscultated in 4 GPs. Reflex testing (FWR, IG) led to a short term ReR increase with all three anaesthesias.

### Blood glucose

Mean BG values increased during the use of all three anaesthetic agents. MMF led to the largest BG increase (especially during induction), followed by KX. Iso increased the BG only modestly (Table 6). All acquired BG values remained within physiological ranges (5-16mmol/L, 89-287mg/dL, [6]).

### Reflexes and observations

**Fig 7:**
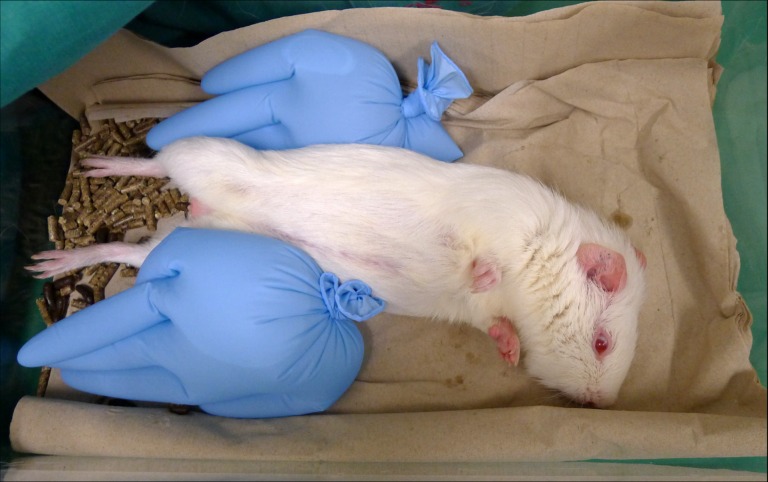
Recovery from ketamine-xylazine anaesthesia in a guinea pig. Cataleptic male guinea pig during recovery after partial ketamine-xylazine anaesthesia antagonization with atipamezole with warming gloves to compensate hypothermia.

With KX, only 7/13 GPs became surgically tolerant, but those who did fell asleep without displaying excitation. During maintenance they showed an increasing exophthalmos, piloerection and a high muscle tone with sharp reflex responses. After atipamezole the GPs became cataleptic and sedated in dorsal recumbency (Fig 7) and responded only temporarily to reflex testing with twitching and chewing.

It required multiple attempts before RR was achieved and thereafter the GPs showed only a mild thermoregulatory shivering and neck located piloerection. After antagonization the reflex responses varied instead of progressing continuously and the GPs remained sedated long after the return of RR.

Iso exposure led to surgical tolerance in all 13 GPs. During BC induction, the GPs appeared highly stressed (strong defensive reactions for approximately 20sec before slowing and slumping to the bottom of the BC). The skin turned pale and they urinated before losing RR. Throughout the Iso exposure, the skin turned pink, the FWR and IG were weak or delayed with a low muscle tone, but the responses intensified when Iso was stopped and the skin colour normalized. After regaining of RR, multiple GPs showed a hind limbs paresis, although they were walking with their front feet and strong thermoregulatory shivering started, combined with a distinct piloerection.

With MMF 11/13 GPs achieved surgical tolerance. During induction, GPs only displayed excitations when disturbed while falling asleep. Throughout MMF the ear reflex remained ±, the muscle tone was high and FWR and IG were strong. The reflex responses intensified quickly and progressively after antagonization and the GPs abruptly regained their RR, followed by a short hyperactive phase. At induction the skin colour turned pink, then faded to pale during maintenance and turned pink once more at AFN administration before it normalized thereafter. Thermoregulatory shivering and piloerection were pronounced in the first 30min after return of RR.

In all anaesthesias, reflex responses intensified in following order during recovery: FWR and IG, ear reflex, lid reflex, RR. After antagonization or Iso exposure stop, RR and skin colour returned from the head to the back and the GPs displayed chewing from + FWR and IG onwards.

## Discussion

In this study we have acquired continuous radio-telemetry data concerning the effect of MMF, Iso and KX on hemodynamics and other physiological parameters during anaesthesia in male GPs and we have also described anaesthesia specific observations. As expected, all anaesthetics had an effect on the parameters and we saw substantial differences between them.

The difficulty in anaesthetizing GPs is due to a combination of factors. The choice of anaesthetics is limited, as a venous induction in the awake state is not viable or the anaesthetic does not provide for a quick recovery to enable fast body temperature and energy regulation. Also the evaluation of anaesthetic depth requires experience, anaesthesia and species specific knowledge, as reflex responses and reactions to anaesthetics vary between anaesthetic agent, individual and intra-individually, especially with KX [2]. Therefore GPs profit from a quickly reversible anaesthetic, close monitoring during and after anaesthesia and a qualified and experienced anaesthetist.

The anaesthetic should be chosen on the considerations of 1) safety, 2) convenience, 3) reliability of the anaesthetic effects, 4) short, low-stress induction and recovery time, 5) the influence of hemodynamic parameters and 6) quickly adjustable anaesthetic depth.

We chose the three paravenous anaesthetics KX, MMF and Iso as they were the most used in recent studies. Until now, KX has been the most commonly used anaesthesia in GPs, which has been administered in various dosages (25-120mg/kg for ketamine and 0.2-13mg/kg for xylazine), but was often reported to lack reliability and frequently needed additional dosages to achieve satisfactory surgical depth [2, 7]. Iso is the most used inhalational anaesthetic in laboratory animals, due to its good hypnotic and muscle relaxing effect, the minimal metabolization and the quick on- and offset of anaesthesia [8]. It does however require atropine premedication in GPs, because they react to Iso with heavy bronchosecretion, salivation and tear production [9] in addition to the dose-dependent respiratory depression.

MMF is an established anaesthesia in Germany which combines midazolam’s anticonvulsive and sedative action, fentanyl’s strong, short-acting analgesic effect, with medetomidine’s sedative, analgesic and muscle relaxing effects [8]. The single effects are potentiated, enabling a dose reduction of each component and thereby creating fewer side effects. Its benefits are a fast induction, easy extension and a full antagonization with a quick recovery.

### Anaesthesia duration

The 40min anaesthesia duration was chosen as this time span allows the performance of many (surgical) procedures and it allows comparability to past studies [9, 10]. Ideally, the time until reaching the desired anaesthetic depth is short, maintenance duration can be varied as needed and time until regained RR is rapid. A short induction is beneficial as the sensitive excitatory anaesthesia phase II [8] is passed quickly and more anaesthesia time is saved for the planned procedure. Rapid post-anaesthetic return to RR speeds the return to auto-regulation of physiological parameters, especially of BT and BG. All 3 anaesthetics had a short duration until surgical tolerance, but with KX only 7/13 GPs reached an operable reflex depth. Wake-up durations for MMF and Iso were relatively short, such that the GPs’ were able to return to body temperature auto-regulation and normal behaviour quickly after anaesthesia stop.

By comparison, KX, even with partial antagonization, required almost 1h until return of RR and recovery thereafter was unreliable, resulting in the need for continued monitoring. Omitting atipamezole, however, led to yet longer durations until regaining of RR (240min after KX induction in mice, 300min after KX induction in rats; [11]; [12]). In summary, MMF and Iso allow quick and reliable induction until desired anaesthetic depth, a flexible maintenance time and a fast return to stable physiological conditions. By contrast, even partially antagonized KX leads to an undesirably long post-anaesthetic recovery and is therefore not advisable for procedures requiring a reliable recovery.

### Blood pressure

**Fig 8:**
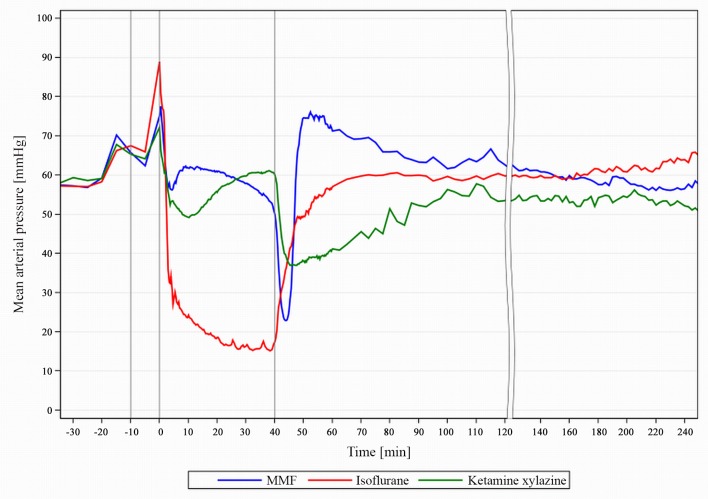
MAP course during MMF, KX and Iso anaesthesia in guinea pigs. Mean arterial blood pressure (MAP) course during anaesthesia in male guinea pigs with medetomidine-midazolam-fentanyl (MMF, n = 11), isoflurane (n = 13) and ketamine-xylazine (n = 7). 1st grey line ≙ premedication at -10min, 2^nd^ line ≙ induction at 0min; 3^rd^ line ≙ end of exposure at 40 min.

The BP guarantees the adequate perfusion of major organs (brain, heart, kidney and liver) and is therefore tightly regulated. Preferably, anaesthesia should have little effect on BP. Our data shows, however, that all 3 anaesthetics influence the BP markedly (Fig 8).

The transient peak of BP associated with induction was most pronounced with Iso due to the comparatively higher handling stress and the defence reactions to the narcotic gas. Iso’s vasodilatory effect led to symptoms of cold feet, pale mucous membranes and weak pulse, which classify as worthy for acute intervention because of risk for vital organ damage [13]. In comparison, rats with Iso only show a mild MAP decrease [10]. Minimal metabolization allows a quick return to a stable MAP after discontinuation in both species.

KX and MMF showed little deviations from baseline MAP, especially MMF had desirable maintenance values. Marked effects are seen in KX and MMF upon administration of atipamezole. In MMF, it reversed the medetomidine hypertonia [14], and we could not observe disadvantageous effects caused by the BP drop. KX produced tolerable BP values until atipamezole induced a moderate hypotension that the GP could only gradually reverse.

Overall, MMF’s effect on BP is acceptable, whereas significant hypotension occurs during Iso maintenance and after partial KX reversal.

### Heart rate

**Fig 9:**
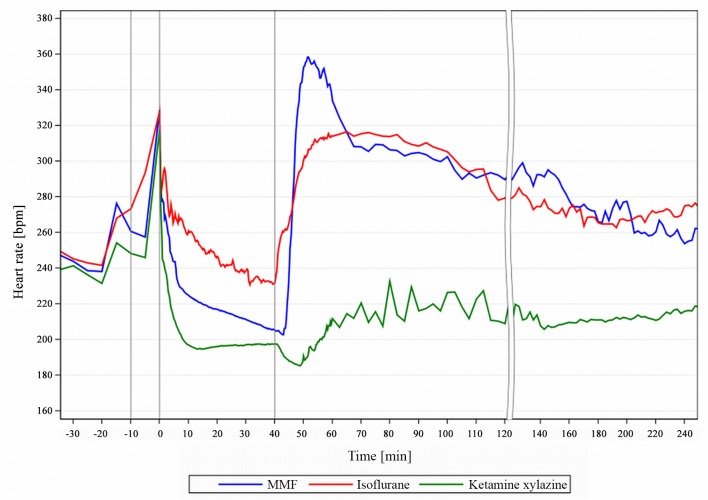
HR course during MMF, KX and Iso anaesthesia in guinea pigs. Heart rate (HR) course during anaesthesia in male guinea pigs with medetomidine-midazolam-fentanyl (MMF, n = 11), isoflurane (n = 13) and ketamine-xylazine (n = 7). 1st grey line ≙ premedication at -10min, 2^nd^ line ≙ induction at 0min; 3^rd^ line ≙ end of exposure at 40 min.

A regular HR ensures constant oxygen supply to vital organs and can also be used to assess anaesthetic depth and analgesia. The transiently increased HR reflects the handling and injection stress and, shortly thereafter, drug-induced effects influence the HR (Fig 9).

With Iso, despite the severe hypotension, GPs displayed only a mild and declining tachycardia, likely due to Iso’s inhibition of the HR baroreceptor reflex [15], which is reversed when Iso is stopped, resulting in a steep increase in HR. The mild bradycardia during MMF maintenance is uncritical and may be the result of the potentiated bradycardic medetomidine effect by fentanyl [16]. The steep HR increase upon antagonization results initially from the atipamezole component in AFN and is intensified after 7.6min by the regained RR (Fig 3), resulting in a transient tachycardia (max. HR 358 bpm at 51.3min, Fig 4D). Similar HR values with Iso and MMF from 70min onwards suggest that HR is no longer under direct pharmacological influence, but may be elevated as a result of thermoregulatory shivering [17].

KX induced a very stable HR from 12.3 until 41min in GPs (Fig 6B and 6C) but with medium bradycardic values, whereas rats showed only a minimally decreased HR with KX [10]. After atipamezole antagonization the HR declines further (-11 bpm, Fig 6C and 6D), despite the drop in pressure which would usually cause a reflex tachycardia as seen with MMF antagonization. The remaining ketamine induces a catalepsy, preventing the RR return and with that a movement-induced HR increase.

In conclusion, Iso produced values closest to physiological HR range, closely followed by MMF. Antagonized KX, however, resulted in an anaesthetic and long term post-anaesthetic bradycardia and should therefore only be considered when a long lasting bradycardia is acceptable, but with substantial effects on other physiological parameters.

### Core body temperature

Anaesthesia often leads to hypothermia, especially in small mammals. Their large body surface-to-volume ratio and absent BT production due to lack of movement during anaesthesia, are amplified by the pharmacological depression of central thermoregulation and by ventilation with cold gases [8].

Hypothermia leads to a slowed metabolism during anaesthesia with a prolonged recovery or even post-anaesthetic death [18].

**Fig 10:**
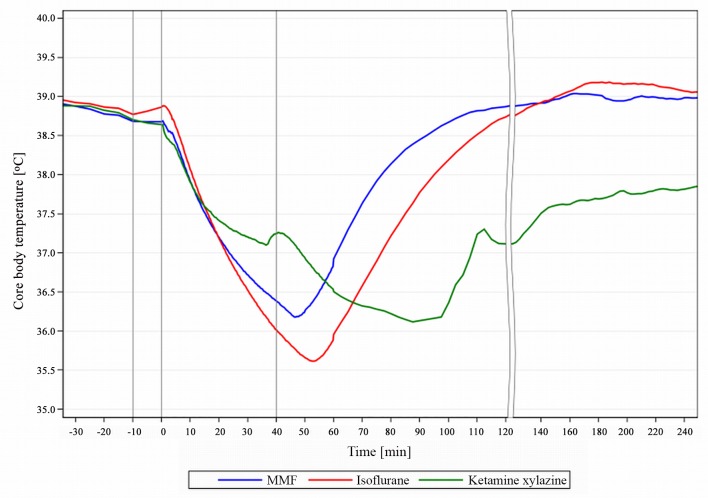
BT course during MMF, KX and Iso anaesthesia in guinea pigs. Core body temperature (BT) course during anaesthesia in male guinea pigs with medetomidine-midazolam-fentanyl (MMF, n = 11), Isoflurane (n = 13) and ketamine-xylazine (n = 7). 1st grey line ≙ premedication at -10min, 2nd line ≙ induction at 0min; 3rd line ≙ end of exposure at 40 min.

We also observed a rapid BT loss, despite the use of water heated mats (temperature losses KX: -2.4°C in 97.5min, Iso: -3.3°C in 53.3min, MMF: -2.5°C in 47min). The time course of the BT loss was similar with all 3 anaesthetic agents up to 15min after induction. BT with MMF decreased until AFN was administered and would likely have continued to decrease, had the anaesthesia not been antagonized (xylazine-fentanyl-climazolam, 33.6°C at 45-90min after induction; [19]). During Iso, BT is lost faster (Fig 10) due to redistribution of body warmth from the body core to the periphery caused by peripheral vascular dilation [20].

We can only hypothesize, why the BT remains the highest at 40min with KX. A combination of insulating factors, namely the high muscle tension, peripheral vasoconstriction and a lower blood flow to the periphery by a reduced heart rate, may result in a slowed BT loss. Further studies are needed to explore the precise mechanism of action of KX on the BT in GPs.

After discontinuation of Iso or antagonization of MMF with AFN, the GPs started thermoregulatory shivering and increased their BT steadily, in contrast to KX, where the catalepsy impeded both shivering and physiological movement. Only 3/7 KX GPs had returned to baseline BT after over 5h. Considering this, the 2 hypothermic GPs would probably have died eventually without additional warming.

Apparently water heated mats can only slow the BT loss during anaesthesia, such that further BT retaining methods like short anaesthesia times and warming of a larger body surface area are required. A close post anaesthetic BT monitoring and optional external warming are especially essential with KX use. In summary, MMF led to the least BT loss and the fastest return to physiological levels, followed closely by Iso, whereas KX led to a prolonged and only slowly reversible hypothermia.

### Respiration

**Fig 11:**
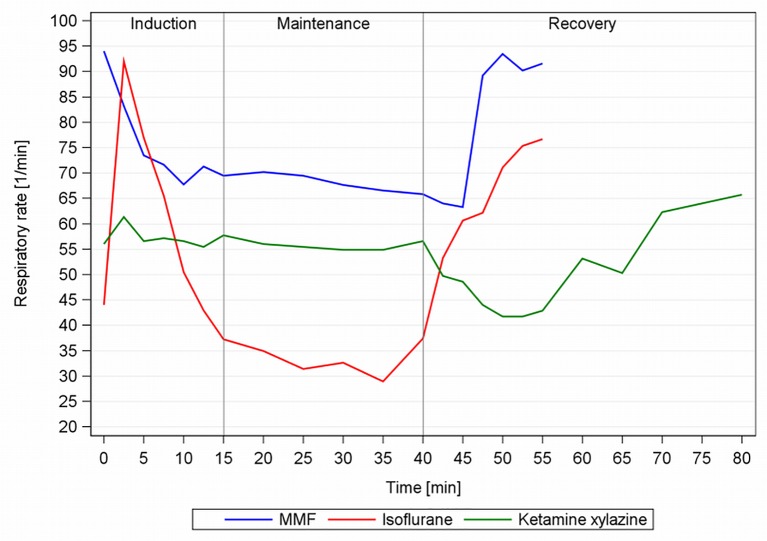
Respiratory rate course during MMF, KX and Iso anaesthesia in male guinea pigs. MMF = medetomidine-midazolam-fentanyl; n = 11, Iso = isoflurane, n = 13; KX = ketamine-xylazine, n = 7. Anaesthesia was lifted after 40min.

GPs often react with hypoventilation to anaesthesia [9], however mechanical ventilation is not recommended as remnant food particles are easily transferred into the lung during intubation [21]. Therefore, reliable spontaneous breathing is crucial. Iso use is especially critical, as the GPs’ long air passages are exposed to Iso’s respiratory depressive, bronchodilatory and hyper-salivatory properties. The water secretion to the saliva in the lower respiratory tract can be transiently reduced by atropine premedication but thick mucous accumulates nevertheless. Comparatively high Iso concentrations are therefore needed to reach the mucous membrane. The GP’s airways and mucous membranes were irritated by Iso, resulting in irregular breathing during induction (Fig 11, [9]).

During maintenance balancing anaesthesia depth and hypoventilation proved challenging and adjustments were often done in 0.1% Iso concentration steps. The decrease in ReR during Iso maintenance of more than 2/3 compared to physiological ReR is partly mediated through enhancement of central GABA_A_ receptor responses [22]. More than 50% ReR decrease compared to physiological values indicates impending respiratory failure and the need for treatment [23], which we observed during Iso and post-anaesthetic KX. We supplemented O_2_ to prevent hypoxia and used the lowest Iso concentrations possible, but lengthy Iso anaesthesia with the observed hypotension and respiratory depression could lead to hypoxic tissue damage.

The hypoventilation during KX is the result of the potentiation of ketamine’s respiratory depression by xylazine [24]. In the recovery phase (Fig 11) the respiratory stimulating atipamezole effect is overcome by the high muscle tension and sedation, which impede physiological ReR.

With MMF, the medetomidine induced hypoventilation is potentiated by fentanyl, as action potential transmission from the pre-Bötzinger complex in the ventrolateral medulla is inhibited, leading to skipped inspirations and irregular breathing [25]. Xylazine-fentanyl-climazolam anaesthesia in GPs led to a similar but slightly lower ReR decrease (52 brpm, [19]) compared to MMF, suggesting that xylazine depresses ReR more than medetomidine.

However, the potentiation of the single components with MMF and thereby achieved component dose reduction, avoids severer respiratory side effects, making it the anaesthesia of choice concerning effects on respiration.

### Blood glucose

**Fig 12:**
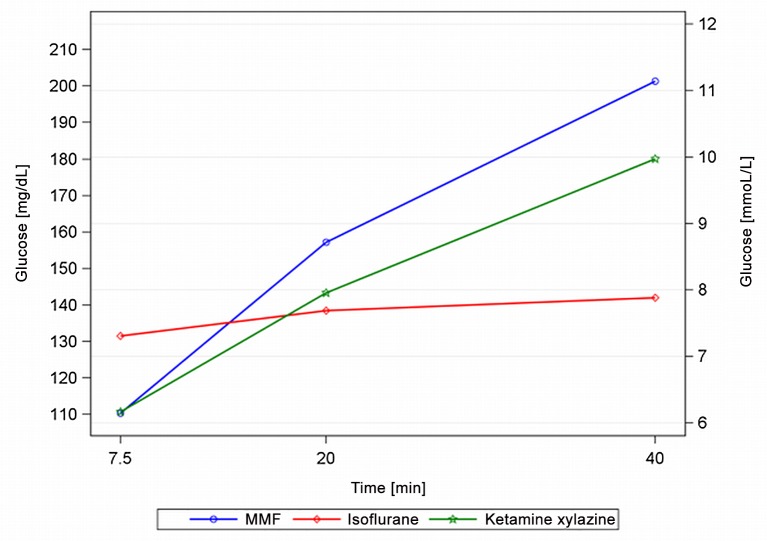
Blood glucose during MMF, KX and Iso anaesthesia in male guinea pigs. MMF = medetomidine-midazolam-fentanyl; n = 11, Iso = isoflurane, n = 13; KX = ketamine-xylazine, n = 7.

A BG increase can be induced by acute stress or the anaesthetic agent and values need to be interpreted accordingly. In a preliminary study pre-anaesthetic BG measurements were attempted in awake GPs, but this led to severe defence reactions and resulted in delayed anaesthesia induction. Therefore, BG tests in awake GPs were not performed in this study. Although GPs are easily stressed, catecholamine-induced hepatic glycogenolysis probably did not cause the rise (Fig 12) with MMF and KX, as BG levels were tested for the first time after loss of consciousness and α_2_-adrenoceptor agonists reduce catecholamine levels.

However, comparative stress-hormone blood levels and pre-anaesthetic BG are needed to confirm this hypothesis. Looking at pharmacological effects, xylazine inhibits the insulin secretion on the β-cells in the pancreas [26] and ACTH and corticosterone are decreased. Also, plasma glucagon and growth hormone levels increase during KX [27]. Therefore, the rise in BG with KX likely originates from an insulin shortage, reducing the uptake of glucose into the cells and an increased hepatic glucose production and secretion.

Concerning MMF, medetomidine induces hyperglycaemia, which could be lowered by adding midazolam in a cat model [26]. Fentanyl passively increases the BG level by inhibiting glucose-stimulated insulin release, which was seen in rat pancreatic islet cells [28].

The greater BG increase in MMF compared to KX (see Table 6) could be explained by medetomidine’s higher α_2_-selectivity (1620 medetomidine:160 xylazine (Virtanen, 1988)). Clinically, GPs with MMF are able to compensate for the BG increase with a quick return to food consumption 30min after AFN administration [18]. BG measurements after anaesthesia are needed to assess and interpret the post-anaesthetic development. Concerning our BG data, Iso produced the smallest increase but all values were within the physiological range, so no anaesthetic can be clearly favoured.

### Reflexes and observations

The assessment of reflex responses is an essential tool to evaluate anaesthesia depth and phase and should be performed regularly by the same person and in combination with evaluation of ReR and HR. The RR marks a successful induction and completes the wake-up phase and the FWR and the IG allow a conclusive assessment of surgical tolerance in the GP [19, 29]. Stress- and painful stimuli (repeated reflex testing, loud noises, touching) during induction should be avoided as they prolong the time until loss of RR. Testing for FWR, instead of the interdigital reflex examines a deeper consciousness disconnection via pain character (bone pain vs. skin pain) and it allows simultaneous muscle tone assessment. Species and anaesthetic agent specific knowledge is essential for the adequate interpretation of reflex responses. We, like Seidensticker [18], observed anaesthesia-related differences in reflex response character and speed. Whereas reflex responses were almost exaggerated during MMF anaesthesia, they were distinct but comparatively reduced with KX and generally slow and weak under Iso anaesthesia. We chose an anaesthetic depth with a mildly positive FWR and IG, as a complete response loss leaves ReR as the only indicator, which is especially questionable with Iso. It also removes the assessment of the dose safety margin until death of the animal. Iso’s effect of delayed and weak reflex responses, combined with an observed transient hind limb paresis directly after regaining of RR (also reported by Heide [9]), made us question the significance of FWR and IG for anaesthetic depth evaluation during Iso.

Under Iso, motoneuronal excitability in the spinal cord is suppressed [30], which may play an important role for the surgical immobility property. As GPs regain motor function after anaesthesia from head to back, the altered synaptic transmission could explain the temporary muscle paresis. With KX the anaesthesia was unreliable in its induction, achieving and in the duration of a surgical plane (only 7/13 GPs achieved surgical tolerance). The GPs needed intensive monitoring after antagonization, with reduced behaviour and abnormal posture even after return to RR.

MMF administration in GPs was rapid, tolerated well and the reflex responses were predictable and consistent during maintenance. After AFN the GPs returned quickly to pre-anaesthetic behaviour and posture. As AFN also reverses fentanyl, painful procedures need to be treated with NSAIDs or metamizole following the anaesthesia to provide adequate analgesia. For the sake of this study, reflex responses were tested every 2.5 min during induction, but this should be omitted in the standard setting, as it causes an unnecessary disturbance without adding beneficial information. Instead, we suggest monitoring for the loss of RR and assessing the FWR and IG for the first time 2min thereafter. Also premedication for MMF and KX was only necessary for comparison in this study, so this stressor can normally also be eliminated.

MMF was the pre- and post-surgically least stressful choice for the animal and allowed reliable insights concerning anaesthetic depth and stage for the anaesthetist.

## Conclusion

Based on our data, MMF is the anaesthesia of choice in GPs for any procedure that exceeds a short, not painful immobilization, for which Iso would be a possible option. HR influence was acceptable and BP, BT, ReR impact, reflex responses and the low anaesthesia related stress were more beneficial for the GP compared to Iso and KX. Iso offered the benefits of quick induction and reliable anaesthesia and could therefore be used for short and not painful procedures (blood withdrawal, examination). However, dramatically low BP and ReR, as well as fast BT loss and uncertain reflex responses make it undesirable for procedures longer than 10min.

KX results were not satisfactory during anaesthesia and disadvantageous in the post-anaesthetic phase, which is why we advise against KX use in GPs.
